# Lymphatic Node Metastasis Risk Scoring System: A Novel Instrument for Predicting Lymph Node Metastasis After Thymic Epithelial Tumor Resection

**DOI:** 10.1245/s10434-021-10602-0

**Published:** 2021-08-27

**Authors:** Xinxin Cheng, Yaxin Lu, Sai Chen, Weilin Yang, Bo Xu, Jianyong Zou, Zhenguang Chen

**Affiliations:** 1 Present Address: Department of Cardiothoracic Surgery of East Division, The First Affiliated Hospital of Sun Yat-Sen University, Guangzhou, Guangdong People’s Republic of China; 2 Department of Clinical Data Center, The Third Affiliated Hospital of Sun Yat-Sen University, Guangzhou, People’s Republic of China; 3 Department of Center for Private Medical Service and Healthcare, The First Affiliated Hospital of Sun Yat-Sen University, Guangzhou, People’s Republic of China; 4 Present Address: Department of Thoracic Surgery, The First Affiliated Hospital of Sun Yat-Sen University, Guangzhou, Guangdong People’s Republic of China

## Abstract

**Background:**

The authors aimed to create a novel model to predict lymphatic metastasis in thymic epithelial tumors.

**Methods:**

Data of 1018 patients were collected from the Surveillance, Epidemiology, and End Results database from 2004 to 2015. To construct a nomogram, the least absolute shrinkage and selection operator (LASSO) regression model was used to select candidate features of the training cohort from 2004 to 2013. A simple model called the Lymphatic Node Metastasis Risk Scoring System (LNMRS) was constructed to predict lymphatic metastasis. Using patients from 2014 to 2015 as the validation cohort, the predictive performance of the model was determined by receiver operating characteristic (ROC) curves.

**Results:**

The LASSO regression model showed that age, extension, and histology type were significantly associated with lymph node metastasis, which were used to construct the nomogram. Through analysis of the area under the curve (AUC), the nomogram achieved a AUC value of 0.80 (95 % confidence interval [Cl] 0.75–0.85) in the training cohort and 0.82 (95 % Cl 0.70–0.93) in the validation cohort, and had closed calibration curves. Based on the nomogram, the authors constructed the LNMRS model, which had an AUC of 0.80 (95 % Cl 0.75–0.85) in the training cohort and 0.82 (95% Cl 0.70–0.93) in the validation cohort. The ROC curves indicated that the LNMRS had excellent predictive performance for lymph node metastasis.

**Conclusion:**

This study established a nomogram for predicting lymph node metastasis. The LNMRS model, constructed to predict lymphatic involvement of patients, was more convenient than the nomogram.

**Supplementary Information:**

The online version contains supplementary material available at 10.1245/s10434-021-10602-0.

Thymic epithelial tumors are prevalent in the anterior mediastinum, accounting for approximately 43 % of anterior mediastinal masses.^1^ Lymph node metastasis with invasion of adjacent organs was found to occur more frequently than lymph node metastasis without such invasion, and the findings showed that the frequency of lymphatic metastasis far exceeds that of previous empirical knowledge.^2,3^

Lymph node metastasis is an important factor affecting tumor recurrence and the prognosis of patients with common tumors, and lymphadenectomy can be performed to accurately stage the tumor and control disease progression.^4–6^ Unfortunately, lymph node examination currently is less frequently performed during thymectomy, which can increase the likelihood that the degree of disease progression in patients will be misinterpreted. Therefore, an urgent need exists for a model to predict lymph node metastasis in thymic epithelial tumors to assist in clinical diagnosis and treatment.

Predictive models for lymph node metastasis have been developed for many cancer types, such as squamous non-small cell lung cancer, esophageal squamous cell carcinoma, colorectal cancer, and so on.^7–9^ Nevertheless, a model for predicting lymphatic involvement of thymic epithelial tumors is hard to construct. The construction of a prediction model faces two main challenges. First, the incidence of the disease is low. The overall incidence of thymoma is 0.13 per 100,000 person-years in America and^10^ 0.09 to 0.23 per 100,000 person-years in Europe.^11^ Second, no lymph node map for thymic epithelial tumors has existed in the past as a public reference for lymphatic resection of thymic epithelial tumors. Therefore, a new lymph node map was proposed by the International Thymic Malignancy Interest Group (ITMIG) and published in the 8th edition of tumor-node-metastasis (TNM) stage classification system for thymic malignancies.^12,13^

Using the Surveillance, Epidemiology, and End Results (SEER) database, this study aimed to develop and validate a predictive model for lymph node metastasis status after thymic epithelial tumor resection. The results of this study can be conveniently implemented in clinical work and can contribute to further guidance and optimization of treatment strategies for thymic epithelial tumors.

## Materials and Methods

### Patients and Study Design

The population data on thymic epithelial tumors were extracted from the SEER database of the American National Cancer Institute. The Incidence‐SEER 18 Regs Research database is based on the November 2017 submission through SEER*Stat software version 8.3.6 (Information Management Services, Inc., Calverton, MD).

**Fig. 1:**
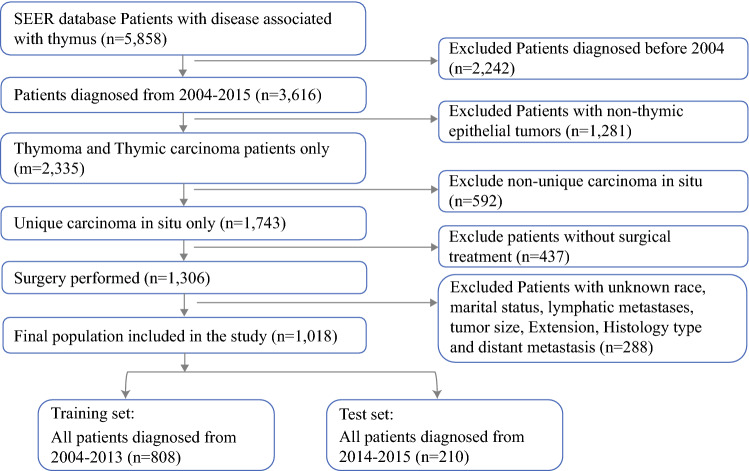
Population inclusion flowchart

As shown in Fig. 1, patients with a diagnosis of thymic epithelial tumors between 2004 and 2015 were selected for the study from the SEER database for public use. All population data were used to divide the patients into two cohorts. The patients who received surgery between 2004 and 2013 formed the training cohort, and those who received surgery between 2014 and 2015 formed the validation cohort.

The inclusion criteria specified the following: (1) The histopathologic diagnosis had to be included. All data had to be histologic type ICD-O-3, and the histologic type had to be according to the International Classification of Diseases for Oncology, third revision (ICD-O-3) using the codes according to the 2015 World Health Organization Classification of Tumors of the Thymus.^14^ The codes for thymic carcinoma were 8004, 8011, 8020, 8021, 8032, 8033, 8052, 8070, 8071, 8072, 8073, 8074, 8075, 8082, 8083, 8090, 8094, 8123, 8140, 8260, 8310, 8430, 8480, 8481, 8560, 8575, 8576, 8586, 8588, 8589, and 8980, and the codes for thymoma were 8580, 8581, 8582, 8583,8584, All the histology type ICD codes were accompanied with the malignant behavior code-3. (2) Only patients with a diagnosis of tumor were included. (3) Only patients who received surgery were included.

The exclusion criteria ruled out patients whose race, marital status, lymphatic metastases, tumor size, tumor extension, histology type, or distant metastasis was unknown.

### Variable Definition

The candidate variables in the analysis were age at diagnosis, sex, race, marital status, tumor size, tumor extension, histologic type, and histologic grade. Race was separated into white, black, and Asian (Asian Indian, Pakistani, Chinese, Filipino, Japanese, Kampuchean, Korean, Laotian, and Vietnamese). Marital status was grouped as single (divorced, separated, single, or unmarried or domestic partner) and married. Extension of tumor included four subgroups: location (CS Extension code 100 or 300 and CS Mets at Dx code 00 or 10), adjacent connective tissue (CS Extension code 400 and CS Mets at Dx code 00 or 10), adjacent organs/structures (CS Extension code 600 and CS Mets at Dx code 00 or 10), and distance (two states according to the SEER manual: (1) CS Extension code 100, 300, 400, or 600 and CS Mets at Dx code 40 or 50 and (2) CS Extension code 800).^15^ Thymic epithelial tumors were classified into low-risk thymomas (type A, AB, and B1), high-risk thymomas (type B2 and B3), and thymic carcinomas (type C).^16^

### Statistical Analysis

Continuous data are described using median (interquartile range [IQR), and categorical data are described as counts and percentages. Least absolute shrinkage and selection operator (LASSO) regression were performed on the training cohort using the lars package (https://mirrors.tuna.tsinghua.edu.cn/CRAN/web/packages/lars/lars.pdf), and three unsparse variables were finally retained for inclusion in the final prediction model after feature selection.^17^ Nomograms were plotted for visual analysis by using the rms package of R.^18^

To decrease overfit bias, we used area under receiver operating characteristic curve (AUC) and calibration with 1000 bootstrap samples to measure the predictive performance of the nomogram. For convenience of clinical use, a novel scoring model was established, which could make clinical prediction easier and more convenient. To estimate the performance of the scoring model, we used AUC, sensitivity, specificity, and accuracy. All statistical test results were considered significant when *p* was lower than 0.05. All statistical analyses were performed in R-3.6.2 (R Foundation for Statistical Computing, Vienna, Austria).^19^

## Results

### Patients Characteristic

As shown in Table 1, the statistical analysis included 1018 eligible patients divided into a training cohort (808 patients) and a validation cohort (210 patients). Men accounted for about a half of the cohorts (52.4 %), and the median age was 59.0 years (range, 48.0–67.0 years). The median tumor size was 64 mm (range, 45.0–89.8 mm), and local invasion was mainly tumor invasive extension (35 %). Thymoma accounted for more of the thymic epithelial tumors (74.8 %), and the majority were low-risk thymoma (40.5 %). Lymph node metastasis was found in 9.5 % of the study cohort.

**Table 1:** Characteristics of patients in the training and validation groups

Variables	Overall cohort *n* (%)	Training cohort (*n* = 808)		Validation cohort (*n* = 210)	
		Metastasis *n* (%)	No metastasis *n* (%)	Metastasis *n* (%)	No metastasis *n* (%)
No. of patients	1018 (100.0)	82 (10.1)	726 (89.9)	15 (7.1)	195 (92.9)
Age: years (range)	59.0 (48.0–67.0)	60.0 (51.0–72.0)	58.0 (48.0–66.0)	59.0 (47.5–66.0)	60.0 (48.0–67.0)
Sex					
Male	533 (52.4)	48 (58.5)	370 (51.0)	13 (86.7)	102 (52.3)
Female	485 (47.6)	34 (41.5)	356 (49.0)	2 (13.3)	93 (47.7)
Race					
White	722 (70.9)	63 (76.8)	513 (70.7)	8 (53.3)	138 (70.8)
Black	132 (13.0)	11 (13.4)	94 (12.9)	4 (26.7)	23 (11.8)
Asia	164 (16.1)	8 (9.76)	119 (16.4)	3 (20.0)	34 (17.4)
Marital status					
Married	653 (64.1)	49 (59.8)	466 (64.2)	11 (73.3)	127 (65.1)
Single	365 (35.9)	33 (40.2)	260 (35.8)	4 (26.7)	68 (34.9)
Tumor size: mm (range)	64.0 (45.0–89.8)	65.5 (46.2–80.0)	65.0 (47.2–90.0)	75.0 (51.0–98.5)	60.0 (40.0–85.0)
Extension					
Localization	356 (35.0)	13 (15.9)	262 (36.1)	1 (6.67)	80 (41.0)
Adjacent connective tissue	216 (21.2)	9 (11.0)	158 (21.8)	2 (13.3)	47 (24.1)
Adjacent organs/structures	315 (30.9)	34 (41.5)	230 (31.7)	6 (40.0)	45 (23.1)
Distance	131 (12.9)	26 (31.7)	76 (10.5)	6 (40.0)	23 (11.8)
Histology type					
Low-risk group	412 (40.5)	12 (14.6)	304 (41.9)	2 (13.3)	94 (48.2)
High-risk group	349 (34.3)	16 (19.5)	267 (36.8)	3 (20.0)	63 (32.3)
Thymic carcinoma	257 (25.2)	54 (65.9)	155 (21.3)	10 (66.7)	38 (19.5)
Histologic grade					
G1	61 (5.99)	5 (6.10)	49 (6.75)	0 (0.00)	7 (3.59)
G2	41 (4.03)	3 (3.66)	31 (4.27)	1 (6.67)	6 (3.08)
G3	95 (9.33)	23 (28.0)	52 (7.16)	6 (40.0)	14 (7.18)
G4	25 (2.46)	3 (3.66)	19 (2.62)	1 (6.67)	2 (1.03)
Unknown	796 (78.2)	48 (58.5)	575 (79.2)	7 (46.7)	166 (85.1)
Overall survival (years)					
3	689 (85.3)	52 (63.4)	637 (87.7)	N/A	N/A
5	635 (78.6)	41 (50.0)	594 (81.8)	N/A	N/A
10	599 (74.1)	36 (43.9)	563 (77.5)	N/A	N/A

N/A: The validation set was not available for a prolonged enough follow-up period, resulting in a survival rate that was not applicable

### Feature Selection Based on LASSO

**Fig. 2:**
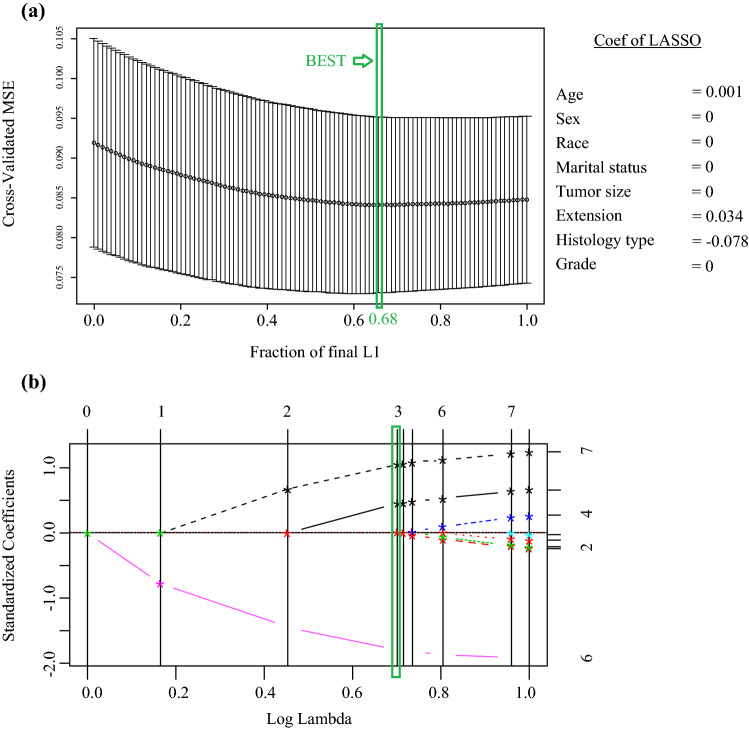
Feature selection using least absolute shrinkage and the selection operator (LASSO) regression model

By running least absolute shrinkage and selection operator (LASSO) regression analyses, according to 10-fold cross-validation, a lambda (*λ*) value of 4.79 with a log (*λ*) of 0.68 were chosen (1-SE criteria), and features with non-zero coefficients were filtrated as the risk factors of thymic epithelial tumor involvement, as shown in Fig. 2. From eight features, this study selected three: age, extension, and histology type.

### Construction of the Prognostic Model

**Fig. 3:**
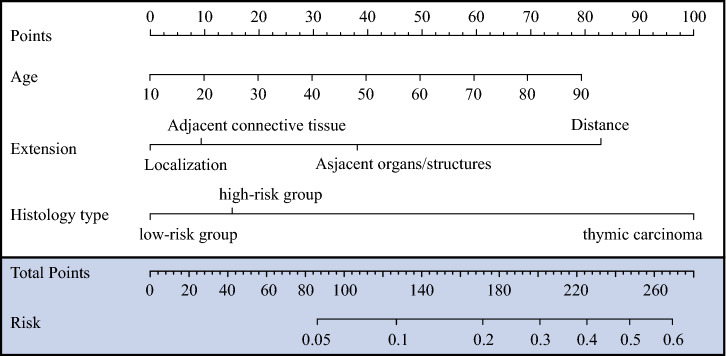
Lymph node metastasis of thymic epithelial tumor nomogram

**Fig. 4:**
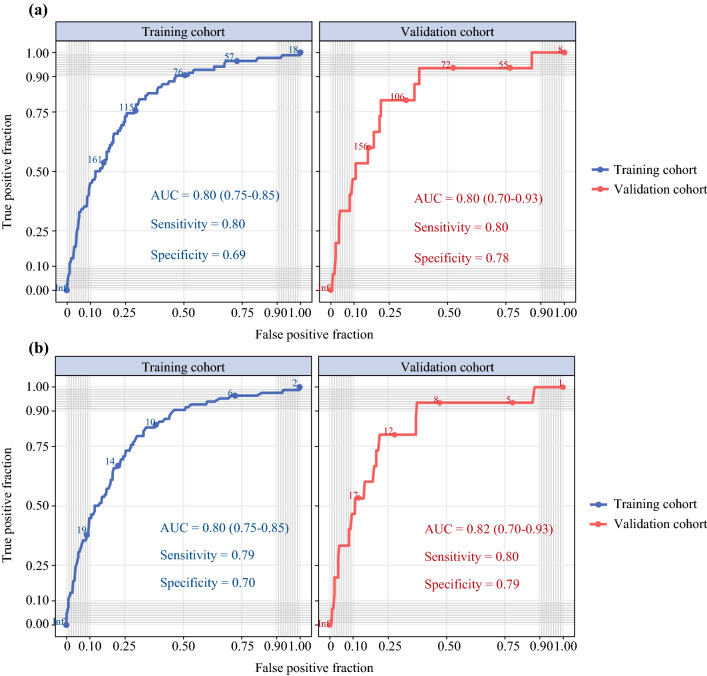
Receiver operating characteristic (ROC) curves for lymph node metastasis prediction for patients using **a** the nomogram and **b** the Lymphatic Node Metastasis Risk Scoring System (LNMRS)

As shown in Fig. 3, a nomogram was established based on feature selection. The predicted AUC of the nomogram was 0.80 (95 % confidence interval [CI] 0.75–0.85) for the training cohort, and 0.82 (95 % CI 0.70–0.93) for the validation cohort, as shown in Fig. 4A. Detailed scores of all the variables in the nomogram are shown in Table 2.

**Table 2:** Detailed scores of each predictor in the nomogram and the LNMRS

Variables	Nomogram point	LNMRS point
Age	1.008*Age-10	0.1*Age-1
Extension		
Localization	0	0
Adjacent connective tissue	9	1
Adjacent organs/structures	38	4
Distance	83	8
Histology type		
Low-risk group	0	0
High-risk group	15	2
Thymic carcinoma	100	10

*LNMRS* the lymphatic node metastasis risk scoring system

**Fig. 5:**
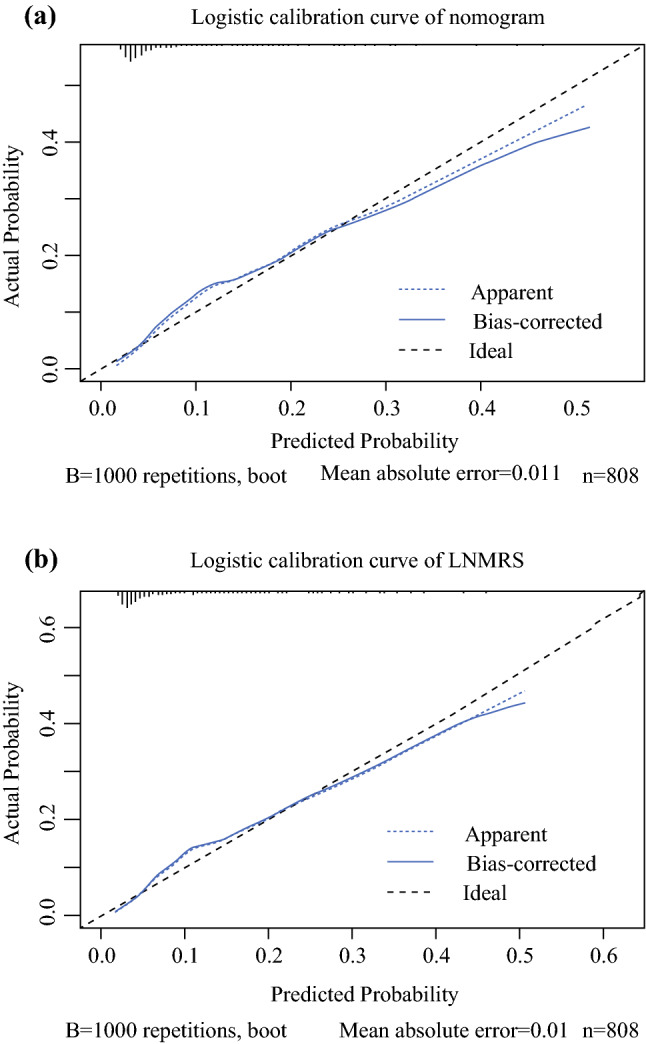
Calibration curves for lymph node metastasis prediction for patients in the training cohort using **a** the nomogram and **b** the Lymphatic Node Metastasis Risk Scoring System (LNMRS)

Based on the score of each variable in the nomogram, a simpler and more generalizable model, called Lymphatic Node Metastasis Risk Scoring System (LNMRS), was constructed, as shown in Table 2. The predicted AUC of the LNMRS was 0.80 (95 % CI 0.75–0.85) for the training cohort, and 0.82 (95 % CI 0.70–0.93) for the validation cohort, as shown in Table 3. The receiver operating characteristic (ROC) curve is shown in Fig. 4B. Meanwhile, detailed scores were calculated, as shown in Table 2. The calibration curves are presented as prediction curves closed to the standard curve, as shown in Fig. 5.

**Table 3:** Model performance in the nomogram and the LNMRS

Evaluation index	Nomogram		LNMRS	
	Training cohort (*n* = 808) *n* (95 % CI)	Validation cohort (*n* = 210) *n* (95 % CI)	Training cohort (*n* = 808) *n* (95 % CI)	Validation cohort (*n* = 210) *n* (95 % CI)
AUC	0.80 (0.75–0.85)	0.82 (0.70–0.93)	0.80 (0.75–0.85)	0.82 (0.70–0.93)
Sensitivity	0.80 (0.72–0.89)	0.80 (0.60–1.00)	0.79 (0.70–0.88)	0.80 (0.60–1.00)
Specificity	0.69 (0.66–0.72)	0.78 (0.73–0.84)	0.70 (0.67–0.73)	0.79 (0.73–0.85)
Accuracy	0.70 (0.70–0.70)	0.79 (0.78–0.79)	0.71 (0.71–0.71)	0.79 (0.79–0.79)

*LNMRS* the lymphatic node metastasis risk scoring system, *CI* confidence interval, *AUC* area under the curve

**Fig. 6:**
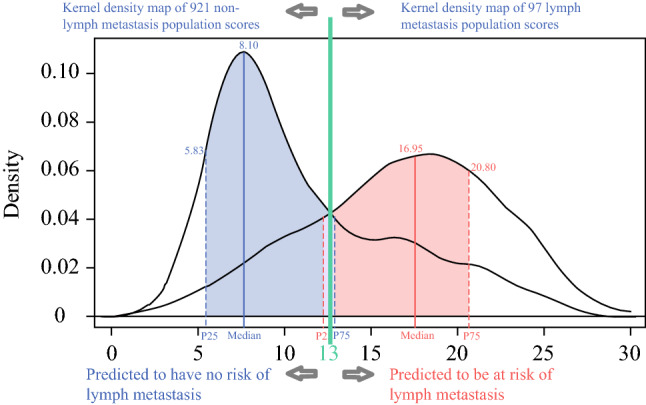
Kernel-density plot of the whole cohort score using the Lymphatic Node Metastasis Risk Scoring System (LNMRS)

We scored the entire cohort population using the LNMRS model and plotted the scores of both cohorts on a kernel-density map based on the incidence of lymph node metastasis. We determined a score of 13 to be the optimal threshold, whereby patients with a score lower than 13 have a low risk of metastasis and those with a score higher than 13 have a high risk of metastasis, as detailed in Fig. 6. For example, if a 40-year-old patient has pathologic thymic carcinoma and an extension of adjacent organs/structures, then this person has an LNMRS score of 17, which indicates a high risk of lymph node metastases based on the kernel-density map.

## Discussion

Currently, no predictive model exists for lymph node metastasis in thymic epithelial tumors. In this study, we developed a simple nomogram-based model called the Lymph Node Metastasis Risk Scoring System (LNMRS), which includes age, tumor extension, and histologic type. This prediction model had an AUC of 0.80 (range, 0.75–0.85) for the training set and an AUC of 0.82 (range, 0.70–0.93) for the validation set, with good discriminative effect and calibration ability. With only three variables, our model was not only objective and accurate, but also easier to generalize to clinical studies.

Some research showed that lymph node status was a significant prognostic factor for patients with thymic epithelial tumors.^2,3,20,21^ Findings suggested that nodal sampling or lymph node dissection can be performed to acquire accurate staging and prediction of prognosis.^2,22^ Our analysis of 1018 patients found that lymphatic metastasis is lymph node metastasis related to age, pathologic type, and tumor extent. This conclusion also was reached in another study.^23^ In addition, we noted patients with negative lymph node findings who had higher postoperative scores and whether some preventive treatment measures, such as adjuvant radiotherapy and individualized postoperative follow-up assessment, could be used for this group of patients.

The National Comprehensive cancer Network (NCCN) suggests that patients with R0 resection need not be treated with chemotherapy or radiotherapy, but should be surveilled for recurrence with an annual chest computed tomography (CT) scan. However, lymph status could not be shown clearly for patients with R0 resection.^24^

In our study, the probability of lymph node metastasis was calculated based on a nomogram with personal clinical information. Patients with R0 resection had high probability of lymphatic metastasis and were more likely to experience lymph node metastasis. Nevertheless, no study exists to support postoperative adjuvant therapy for such patients. Therefore, further research is needed.

The SEER database has a massive amount of clinical information for researchers to perform a large range of clinical studies. Based on the role of lymph node metastasis in disease progression, lymph node prediction using relevant variables from the SEER database has been performed for different malignancies with proven results.^25–27^

However, some limitations of the SEER database are inevitable. First, the SEER database gathers only patients’ clinical information, and neither the consistency nor the standardization of patient treatment could be normalized. Second, the validation set was not available for a sufficiently prolonged follow-up period, resulting in a survival rate that was not applicable. Finally, in the classification of variables, those not described according to the specificity of a different neoplasm (e.g., invasive carcinoma confined to gland of origin in CS Extension) do not distinguish Masaoka stage 1 from stage 2 neoplasms.

## Conclusion

In our research, we developed a new model (LNMRS) for patients with thymic epithelial tumors based on the SEER database. This new model demonstrated perfect performance in predictive accuracy capability. The model could be a useful tool for predicting lymph status in thymic epithelial tumors.

## Supplementary Information

Below is the link to the electronic supplementary material.

Supplementary file1 (JPG 55 kb)
