# Supplementary figures and images for: Lymphatic Node Metastasis Risk Scoring System: A Novel Instrument for Predicting Lymph Node Metastasis After Thymic Epithelial Tumor Resection
Source: Ann Surg Oncol. 2021 Aug 27;29(1):598–605. doi: 10.1245/s10434-021-10602-0 (PMC8677650; doi:10.1245/s10434-021-10602-0)

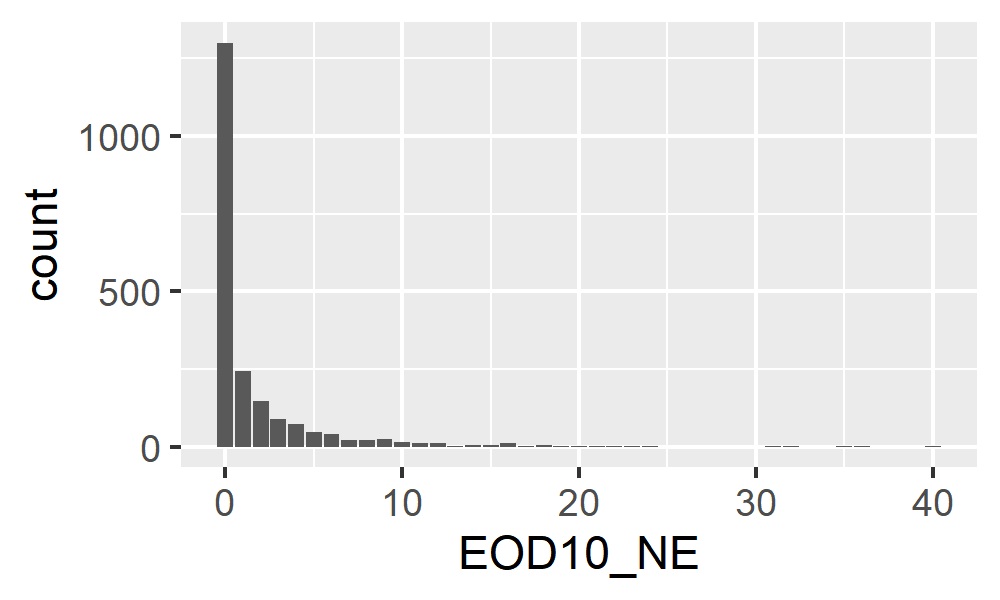

Supplement: Supplementary file 1 — Supplementary file1 (JPG 55 kb) [file 10434_2021_10602_MOESM1_ESM.jpg]
